# Rsp5-mediated ubiquitination of a functional analog of the Rim8 arrestin facilitates Rim pathway activation in *Cryptococcus neoformans*

**DOI:** 10.1128/mbio.00732-25

**Published:** 2025-07-22

**Authors:** Lukas M. du Plooy, Corinna Probst, Matthew E. Capobianco, Charles Giamberardino, Claudia A. Petrucco, J. Andrew Alspaugh

**Affiliations:** 1 Department of Medicine, Duke University School of Medicine 169103, Durham, North Carolina, USA; 2 Department of Molecular Genetics and Microbiology, Duke University School of Medicine 242247 , Durham, North Carolina, USA; 3 Department of Cell Biology, Duke University School of Medicine 207072 , Durham, North Carolina, USA; Yonsei University, Seoul, Republic of Korea

**Keywords:** arrestin, fungal pathogenesis, Rim8, Rim101, alkaline pH response pathway, Rra2, Vps23

## Abstract

Pathogenic microorganisms use varied cellular processes to adapt to the particular stresses encountered in the infected host. These stresses include rapid alterations in ambient temperature, nutrient availability, and extracellular pH. Fungal pathogens, therefore, rely on the activation of stress response pathways such as the Pal/Rim pathway to adapt to the neutral pH encountered when infecting mammals. While this pathway is conserved among human pathogenic fungi, the proteins required for pH sensing appear to have diverged between different fungal phyla. The opportunistic fungal pathogen *Cryptococcus neoformans*, a basidiomycete, employs a pH-sensing protein in its Rim pathway that is distinct but functionally analogous to related proteins in well-studied ascomycete fungal systems. We recently characterized protein ubiquitination mediated through the Rsp5 ubiquitin ligase to be required for *C. neoformans* virulence and for microbial adaptations to host-relevant conditions, including growth at host pH levels. Here, we determined that *C. neoformans* Rsp5 is specifically required for Rim pathway activation. Using an unbiased screen for proteins that are ubiquitinated by Rsp5 in acidic and alkaline pH, we identified a new component of the *C. neoformans* Rim pathway that is targeted by Rsp5 for ubiquitination and shares protein features with the ascomycete Rim8/PalF proteins. Rsp5-mediated ubiquitination facilitates protein interactions with Vps23, a downstream trafficking component of the Pal/Rim pathway. Therefore, we define adaptation to ambient pH as one component of the broad cellular roles of Rsp5-mediated ubiquitination, as we explore how protein ubiquitination affects cryptococcal cell physiology, virulence, and microbial interactions with the host.

**IMPORTANCE:**

Exploring the molecular adaptations allowing fungi to grow in an ever-changing environment yields insight into how fungal pathogens adapt to the stresses present in the infected host. The fungal Rim/Pal pathway, activated during alkaline pH stress and during mammalian infection, is of particular interest because of the lack of a homologous pathway in other eukaryotes, providing an opportunity to identify novel targets for antimicrobial therapies with little damage to the host. There is evidence for convergent evolution in this pathway between ascomycetes and basidiomycetes, evident through the functionally converged, but sequence-dissimilar, sensing proteins found in these two fungal groups. Here, we identify the role of ubiquitination in the activation of the *Cn* Rim pathway. This ubiquitination event is mediated by the Rsp5 E3 Ub ligase and a basidiomycete-specific functional analog of the ascomycete PalF/Rim8 protein that is required for interaction with downstream components of this pathway.

## INTRODUCTION

Many of the same molecular features that favor the survival of fungi in specific environments also allow fungal pathogens to colonize and cause invasive disease in humans. For example, two well-recognized environmental stressors, rapid changes in ambient temperature and pH, are also encountered in an infected mammalian host (1–6). These ever-changing stress conditions in diverse environments, as well as local competition with other microorganisms, provide selective pressures that lead to adaptation to the environment and to conditions encountered during mammalian infection (7–11).

The Pal/Rim pathway is a fungal-specific signaling cascade that is activated in response to alkaline ambient pH (12). This pathway allows fungi to adapt to elevated pH levels as well as in the presence of high cation concentrations, such as sodium and potassium ions encountered in alkaline soil (12). Activation of this pathway during infection is also required for proper organization of cell wall components to allow for evasion of recognition by host Toll-like and C-type lectin receptors, making the Rim signaling relevant to the pathogenesis of fungal pathogens of animals (13, 14). The Pal/Rim pathway includes seven-transmembrane sensor proteins that, upon activation, initiate a cascading series of protein interactions that eventually result in the assembly of a multiprotein proteolysis complex (reviewed in reference 15). The proteolysis complex binds and cleaves the terminal transcription factor in this signaling cascade: Rim101 in yeast-like fungi and PacC in hyphal fungi. Cleavage most likely occurs at the PEST site around 100 residues from the C-terminal end (16, 17). Once cleaved, this transcriptional regulator re-localizes to the nucleus where it controls the expression of genes that favor growth at elevated pH, such as the *CIG1* gene expressing a mannoprotein involved in iron chelation (18).

Many of the components required for the Pal/Rim pathway signal propagation are highly conserved among fungi. These include the Rim101/PacC transcription factor, the members of the proteolysis complex, and trafficking proteins in the endosomal sorting complexes required for transport (ESCRT). However, the proteins involved in pH sensing at the cell surface have diverged between fungal phyla. The pH-sensing Rim21/PalH proteins are highly conserved among ascomycetes, including model yeasts such as *Saccharomyces cerevisiae* (*Sc*) and model molds such as *Aspergillus nidulans* (*An*) (19, 20). These pH sensors contain seven predicted transmembrane domains anchoring them within membranes, as well as extended C-terminal cytoplasmic tails that likely serve to interact with downstream effectors. Interestingly, genes encoding sequence homologs of Rim21/PalH are not present in the genomes of fungi in the phylum Basidiomycota, a large and heterogeneous group of fungi that includes mushrooms as well as plant and animal pathogens. In contrast, basidiomycetes use a structurally similar but sequence-divergent functional ortholog as a surface pH sensor, represented by the Rra1 proteins in *Cryptococcus neoformans* (*Cn*) (21) and *Malassezia sympodialis* (22).

In ascomycetes, proteins linking the surface pH sensors with downstream Rim/Pal pathway components include the Rim8/PalF α-arrestin protein (17, 23). The α-arrestin proteins are often ubiquitinated by the Rsp5 ubiquitin ligase and serve as adaptors to assist in interactions between Rsp5 and other ubiquitination targets (24). The Rim8/PalF α-arrestin is mono-ubiquitinated by the Rsp5 E3 ubiquitin ligase, a modification required for interaction with downstream components of the Rim pathway (25). Ubiquitination of the *Sc* Rim8 and *An* PalF arrestin proteins facilitates interaction with the Vps23/ESCRT-I protein, eventually resulting in the assembly of the Rim20/PalA and Rim13/PalB protein complex required for cleavage and activation of the Rim101/PacC transcription factor (26, 27).

We recently characterized the role of Rsp5-mediated ubiquitination in the metabolism and pathogenesis of the opportunistic fungal pathogen *C. neoformans* (28). This fungus is responsible for around 120,000 deaths per year, predominantly in immunocompromised individuals, such as those living with HIV/AIDS or receiving chronic immunosuppressive therapy (29). The *C. neoformans rsp5*Δ loss-of-function mutant strain has pleiomorphic stress-sensitive phenotypes, including defects in thermotolerance as well as increased susceptibility to high salt and high pH (28). These latter two phenotypes are shared with strains with mutations in the *C. neoformans* Rim alkaline response pathway. We therefore hypothesized that Rsp5 might assist in Rim pathway activation in this human fungal pathogen, as does its ortholog in the model yeast *S. cerevisiae*, despite the apparent basidiomycete-wide loss of the Rim8/PalF arrestin, a target of the Rsp5 ubiquitin ligase in this pathway. We therefore explore the role of *C. neoformans* Rsp5 in known fungal alkaline response pathways, and we characterize a new Rsp5 target protein that mediates Rim pathway activation, growth at elevated pH, and microbe-host interactions.

## RESULTS

### The *Cn* Rsp5 E3 Ub ligase is required for activation of the Rim alkaline pH response pathway

**Fig 1:**
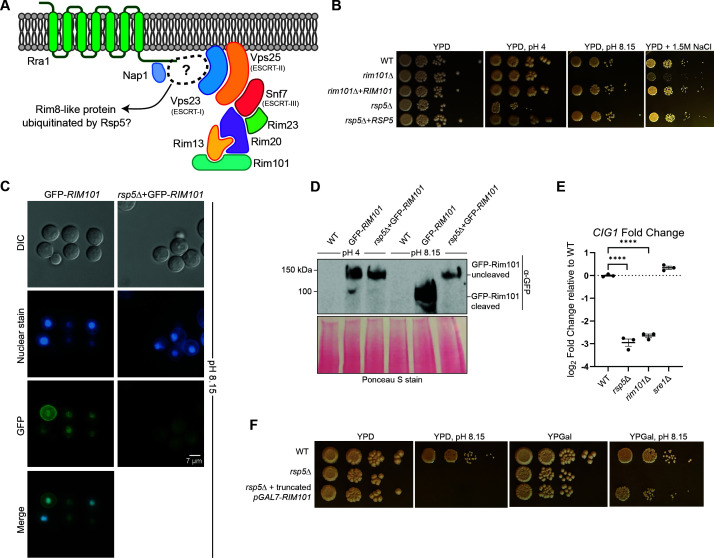
The Rsp5 E3 ubiquitin ligase is required for activation of the *Cryptococcus neoformans* Rim pathway. (**A**) Model of known *Cn* Rim pathway components, including the Rim pathway activation complex (Rra1 surface pH sensor, Nap1 scaffold), the ESCRT-mediated vesicular trafficking complex, and the Rim101 proteolysis complex (Rim23, Rim20, and the Rim13 protease). Unlike the Rim8 arrestin-like protein present in ascomycetes, no known protein has been identified to link the upstream pH sensing components with the downstream pathway effectors in *C. neoformans*. (**B**) Growth phenotypes on acidic, alkaline, and high salt stress plates. Indicated strains were incubated as serial spot dilutions for 72 hours at 30°C on yeast extract-peptone-dextrose (YPD) medium or on YPD medium buffered to pH 4, pH 8.15, or supplemented with 1.5 M NaCl. (**C**) Localization of a green fluorescent protein (GFP)-tagged Rim101 protein was assessed by epifluorescent microscopy in the WT strain or the *rsp5*Δ mutant strain expressing *GFP-Rim101* after 1 hour of incubation in YPD medium at pH 8.15. The location of the nucleus was assessed by co-staining with Hoechst nucleic acid stain. Protein localization was analyzed via microscopy (GFP channel), and images were analyzed via ImageJ/Fiji. Representative differential interference microscopy (DIC), GFP (GFP-Rim101), and DAPI (Hoechst, nuclear) images are shown. (**D**) Alkaline pH-mediated proteolytic processing of the GFP-tagged Rim101 transcription factor. The WT and *rsp5*Δ mutant strains expressing *GFP-RIM101* were incubated in YPD medium buffered to either pH 4 (Rim pathway non-activating conditions) or pH 8.15 (Rim pathway activating conditions) for 1 hour at 30°C prior to trichloroacetic acid (TCA)-based protein extraction. Proteolytic cleavage of GFP-Rim101 was analyzed via western blotting using an anti-GFP antibody (α-GFP), and even protein loading and transfer were assessed via Ponceau S staining. (**E**) Transcript abundance of the Rim101 target gene *CIG1*. Strains were incubated in YPD medium buffered to pH 4 and pH 8.15 for 1 hour at 30°C, and total RNA was harvested. Transcript abundance was assessed by quantitative real-time PCR (qRT-PCR), and the log_2_ fold change relative to WT was calculated using the ΔΔ C_T_ method. Transcript levels were normalized to *GPD1* transcript levels. The mean with error bars indicating the standard error of the mean of three biological replicates is plotted. Statistical analysis was performed using one-way analysis of variance (ANOVA) and an appropriate *ad hoc* test (****P < 0.00001). (**F**) Growth phenotypes of a *rsp5*Δ mutant strain expressing a constitutively active form of Rim101 compared to the *rsp5*Δ mutant strain alone. The indicated strains were incubated as serial spot dilutions for 72 hours at 30°C on YPD medium or on YPD medium buffered to pH 8.15, as well as on medium containing galactose to induce expression of the constitutively active Rim101 protein.

In the model yeast, *S. cerevisiae*, the Rsp5 E3 ubiquitin ligase is required for activation of the Rim alkaline response pathway. One target of this ubiquitin ligase is the Rim8 α-arrestin, for which ubiquitin assists in binding to the ESCRT-I protein Vps23 (25). However, a close homolog of the *Sc* Rim8 protein is absent in *C. neoformans* (Fig. 1A) (21). To determine if the Rsp5 ubiquitin ligase enables activation of this pathway in *C. neoformans*, we incubated the *Cn rsp5*Δ mutant strain in multiple stress conditions to determine if this strain mimics the growth phenotypes of *Cn* Rim pathway mutant strains. Similar to the *Cn rim101*Δ mutant strain, the *rsp5*Δ mutant displayed reduced growth on alkaline media (pH 8.15) and on medium containing 1.5 M NaCl (Fig. 1B). Although activation of the *Cn* Rim pathway starts at pH 6, utilizing medium buffered to a pH of 8.15 allows for optimal distinction of strains with growth defects in alkaline conditions in laboratory settings (21). The *rsp5*Δ mutant strain also demonstrated defects in other stress-responsive phenotypes that are not shared by Rim pathway mutants, including reduced growth at 37°C and in the presence of 0.03% SDS (28). We hypothesized that the phenotypes of the *rsp5*Δ mutant represent a composite effect of defective ubiquitination of multiple target proteins, including those involved in Rim pathway activation. We therefore tested for specific evidence of ineffective Rim pathway activation in the *rsp5*Δ mutant strain.

As previously demonstrated, a green fluorescent protein (GFP)-tagged Rim101 transcription factor re-localizes from the cytoplasm to the nucleus when activated by alkaline pH (21). We therefore used fluorescent microscopy to determine whether GFP-tagged Rim101 demonstrated pH-dependent changes in cellular localization in the *rsp5*Δ mutant. In contrast to wild type (WT), GFP-Rim101 did not demonstrate nuclear enrichment in the *rsp5*Δ strain background when the fungal cells were transferred to yeast extract-peptone-dextrose (YPD) medium, pH 8.15, for 1 hour (Fig. 1C). Since Rim101 nuclear localization is preceded by proteolytic activation, we assessed alterations in GFP-Rim101 by western blot in the WT and *rsp5*Δ mutant at different pH conditions. This study demonstrated alkaline pH-dependent proteolytic processing of GFP-Rim101 in the WT strain and failed proteolysis in the *rsp5*Δ mutant, similar to that observed in other *Cn* Rim pathway mutant strains (Fig. 1D) (21).

We also tested the transcriptional effects of defective Rim101 processing in the *rsp5*Δ mutant by measuring relative transcript levels of the *CIG1* gene, a direct transcriptional target of *Cn* Rim101, and encoding an extracellular mannoprotein involved in iron uptake (30, 31). Transcription of *CIG1* is regulated by the Rim101 transcription factor, and its expression is highly induced at alkaline pH in a Rim101-dependent manner (31). Similar to the expression measured in a *rim101*Δ mutant, *CIG1* transcript levels were not induced under alkaline conditions in the *rsp5*Δ mutant strain (Fig. 1E). In contrast to the case for Rim pathway mutants, *CIG1* expression was induced by alkaline pH in the *sre1*Δ sterol response pathway mutant, a strain with a distinct mechanism of defective alkaline pH growth (Fig. 1E) (32). Finally, we expressed a truncated form of the Rim101 transcription factor in *rsp5*Δ mutant cells lacking 603 amino acid residues at the C-terminal end (21). This truncated form is constitutively active and is able to rescue the growth defect of Rim pathway mutants on alkaline medium, which was observed in the *rsp5*Δ mutant strain expressing truncated Rim101 as well (Fig. 1F). Together, these data demonstrate that *Cn* Rsp5 is required for Rim pathway activation, as assessed by pH-mediated Rim101 proteolytic cleavage and nuclear localization, as well as phenotypic rescue of growth defects on alkaline medium with the expression of constitutively active Rim101 and through assessing the transcriptional regulation of a Rim pathway target gene.

### *Cn* arrestin domain-containing proteins and growth in alkaline pH

In *S. cerevisiae*, Rsp5 mediates Rim pathway activation through its ubiquitinating activity of the Rim8 arrestin-like protein (25). We previously reported that two *Cn* proteins containing both N-terminal and C-terminal arrestin domains (arrestin-like proteins Ali1 and Ali2) are not individually required for growth at pH 8.15, and therefore are not likely required for Rim pathway activation (21). We subsequently identified two additional proteins with a single arrestin domain, Ali3 and Ali4 (28, 33). Ali1, Ali2, Ali3, and Ali4 are the only proteins in the *Cn* genome with classical arrestin domains predicted by simple sequence analysis (33). None of the individual *ali*Δ mutant strains is required for growth at alkaline pH. To rule out functional redundancy among the arrestins, we tested whether a strain lacking all four of the known *Cn* arrestin proteins is able to grow in alkaline pH conditions. The *ali1*Δ *ali2*Δ *ali3*Δ *ali4*Δ quadruple arrestin mutant also grew similarly to WT at pH 8.15 (Fig. S1), indicating that these four arrestin-like proteins are not essential, either individually or in combination, for *Cn* Rim pathway activation and alkaline pH growth.

### Rsp5 does not ubiquitinate known members of the *Cn* Rim pathway

**Fig 2:**
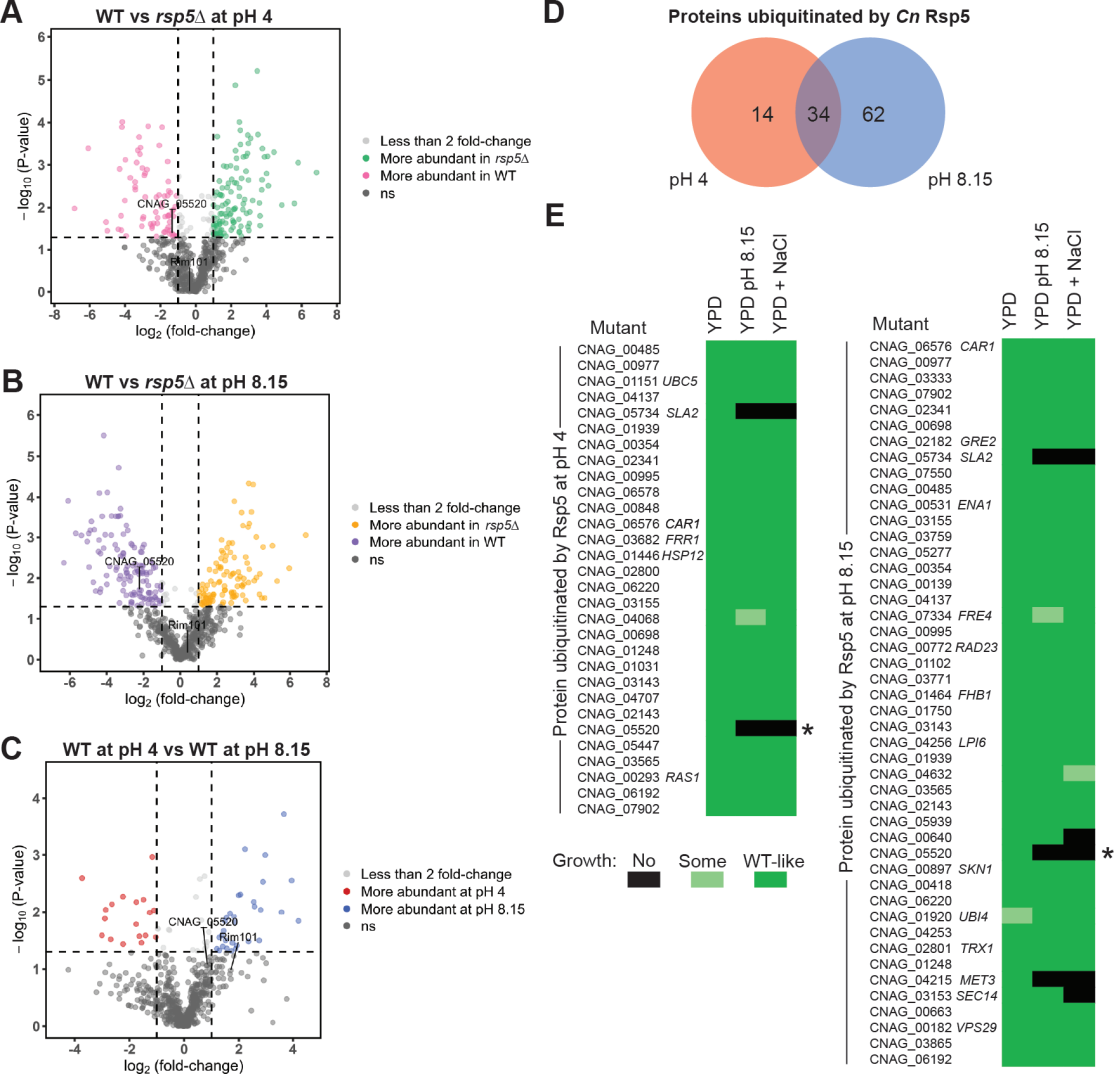
Identification of proteins requiring Rsp5 for ubiquitination at pH 4 and pH 8.15. The WT and *rsp5*Δ strains were incubated in either YPD medium at pH 4 or YPD medium buffered to pH 8.15 for 1 hour at 30°C. Total cell lysates were treated with trypsin, resulting in a ubiquitin remnant stump attached to ubiquitinated protein fragments. These previously ubiquitinated proteins were enriched by immunoaffinity and identified by quantitative liquid chromatography-tandem mass spectrometry. (**A and B**) Volcano plots of individual peptides identified by mass spectrometry demonstrate relative peptide abundance (log_2_ fold change) in the *rsp5*Δ strain compared to WT at either pH 4 (**A**) or pH 8.15 (**B**). (**C**) A volcano plot of individual peptides identified by mass spectroscopy from WT cells with abundance at either pH 4 or at pH 8.15. The identified peptides belonging to the CNAG_05520 and Rim101 proteins are indicated. (**D**) A Venn diagram indicating protein amounts requiring Rsp5 for ubiquitination when exposed to acidic pH or alkaline pH, or that are insensitive to pH. (**E**) A growth phenotype screen using a *Cryptococcus neoformans* mutant strains collection (38) to screen for *rim101*Δ mutant-like stress phenotypes (decreased growth in alkaline pH and high salt media) in mutant strains corresponding to the proteins identified to be ubiquitinated at pH 4 (left) and pH 8.15 (right) in an Rsp5-dependent manner. Black boxes indicate no growth, light green boxes indicate some growth, and dark green boxes indicate WT-like growth. The *CNAG_05520*Δ mutant strain, indicated by an asterisk, was the only strain with decreased *CIG1* expression, as is also seen in a *rim101*Δ mutant strain.

Since no *Cn* proteins with clearly identifiable arrestin domains were required for Rim pathway-related phenotypes, we hypothesized that Rsp5 might mediate Rim pathway activation through a non-canonical adaptor protein. Rsp5 adaptors in other eukaryotes are often ubiquitinated by this E3 Ub ligase as a means for their activation (34, 35). We therefore used a mass spectroscopy-based proteomic method to identify *C. neoformans* proteins that are likely ubiquitinated by *Cn* Rsp5 in a pH-dependent manner. We previously used this experimental protocol to identify *Cn* Rsp5 targets ubiquitinated under high salt conditions (28). We incubated the WT and *rsp5*Δ mutant strains in YPD medium at pH 4 and pH 8.15. Total cell lysates from each condition were digested with trypsin and enriched for ubiquitinated peptides using an antibody recognizing the characteristic “ubiquitin stump” remaining after trypsin treatment (36). Mass spectroscopy of the resulting samples identified 96 proteins that were preferentially ubiquitinated in the WT strain compared to the *rsp5*Δ mutant strain at pH 8.15 and 48 preferentially ubiquitinated in the WT strain compared to the *rsp5*Δ mutant strain at pH 4, while 34 proteins were preferentially ubiquitinated in the WT strain at both pH 4 and pH 8.15 (Fig. 2A through D; Tables S1 and S2). In WT cells, 16 proteins were more abundant in ubiquitinated form at pH 4 than at pH 8.15, and 25 were more abundant at pH 8.15 than at pH 4, although Ub ligases other than Rsp5 might be responsible for their ubiquitination (Fig. 2C; Tables S3 and S4). No known Rim pathway protein was significantly enriched in this group of proteins, although the Rim101 transcription factor did appear in this set as a ubiquitinated protein, consistent with previous findings in *S. cerevisiae* (Fig. 2A through C) (37).

**Fig 3:**
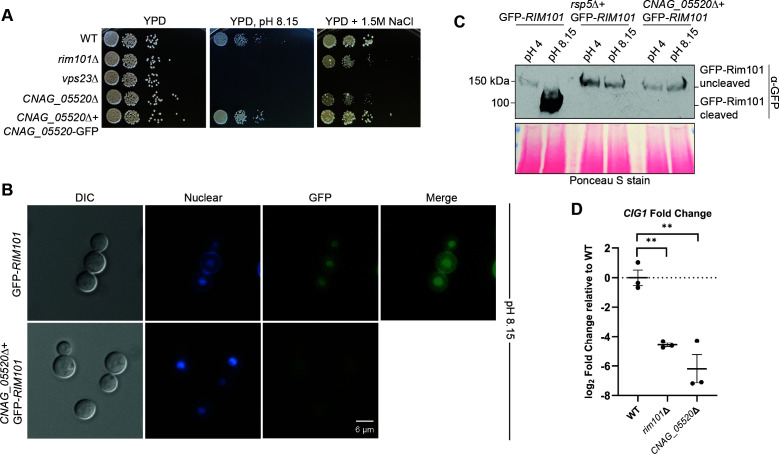
The *CNAG_05520* gene is required for *C. neoformans* Rim pathway activation. (**A**) Growth phenotype on alkaline and high salt stress plates. Indicated strains were incubated as serial spot dilutions for 72 hours at 30°C on YPD medium or on YPD medium either buffered to pH 8.15 or supplemented with 1.5 M NaCl. (**B**) Localization of a GFP-tagged Rim101 protein was assessed by epifluorescent microscopy in the WT strain or the *CNAG_05520*Δ mutant expressing *GFP-Rim101* after 1 hour of incubation in YPD medium at pH 8.15. The location of the nucleus was assessed by co-staining with Hoechst nucleic acid stain. Protein localization was analyzed via microscopy (GFP channel), and images were analyzed via ImageJ/Fiji. Representative DIC, GFP (GFP-Rim101), and DAPI (Hoechst, nuclear) images are shown. (**C**) Alkaline pH-mediated proteolytic processing of the GFP-tagged Rim101 transcription factor. The WT, *rsp5*Δ mutant, and *CNAG_05520*Δ mutant strains expressing *GFP-RIM101* were incubated in YPD medium buffered to either pH 4 (Rim pathway non-activating conditions) or pH 8.15 (Rim pathway activating conditions) for 1 hour at 30°C prior to TCA-based protein extraction. Proteolytic cleavage of GFP-Rim101 was analyzed via western blotting using an anti-GFP antibody (α-GFP), and even protein loading and transfer were assessed via Ponceau staining. (**D**) Transcript abundance of the Rim101 target gene *CIG1*. Indicated strains were incubated in YPD medium, pH 4 or pH 8.15, for 1 hour at 30°C, and total RNA was harvested. Transcript abundance was assessed by qRT-PCR, and the log_2_ fold change relative to WT was calculated using the ΔΔ C_T_ method. Transcript levels were normalized to *GPD1* transcript levels. The mean with error bars indicating the standard error of the mean of three biological replicates is plotted. Statistical analysis was performed using one-way ANOVA and an appropriate *ad hoc* test (**P < 0.001).

To determine if any of the 144 proteins that require Rsp5 for their ubiquitination at either pH 4 or pH 8.15 are required for Rim pathway activation, we made use of an established collection of strains with mutations in non-essential *Cn* genes to screen for Rim pathway-related phenotypes: impaired growth at pH 4 and pH 8.15 was compared for each mutant as well as impaired growth on 1.5 M NaCl (39). Of the 144 differentially ubiquitinated proteins, 76 had a corresponding mutant strain in this collection at the time of this analysis, and more mutants might become available as mutant strain collections are expanded. Three of these strains demonstrated defects in both of these Rim pathway-related stress conditions. However, only one mutant strain, lacking the uncharacterized *CNAG_05520* gene, also demonstrated reduced *CIG1* expression under alkaline conditions similar to Rim pathway mutants (Fig. 3D).

### The *CNAG_05520* gene product is required for activation of the *Cn* Rim101 transcription factor

To determine if the *CNAG_05520*Δ mutant strain, like the *rsp5*Δ mutant strain, is required for Rim101 pathway activation, we assessed alkaline pH and high salt stress growth, Rim101 cleavage and nuclear localization as well as Rim pathway target gene expression in the *CNAG_05520*Δ mutant strain (Fig. 3). Similar to the *rsp5*Δ ubiquitin ligase mutant strain, the GFP-Rim101 protein fails to re-localize in the nucleus in the absence of the *CNAG_05520* gene when cells are incubated in high pH medium (Fig. 3B). Additionally, GFP-Rim101 remains in an uncleaved form in the *CNAG_00520*Δ mutant strain at high pH (Fig. 3C). The functional relevance of failed activation of Rim101 was confirmed by ineffective pH-mediated induction of the Rim101 target gene *CIG1* as determined by quantitative real-time PCR (Fig. 3D).

### Absence of the *CNAG_05520* gene product displays similar pathogenesis-related phenotypes as the *rim101*Δ mutant strain

**Fig 4:**
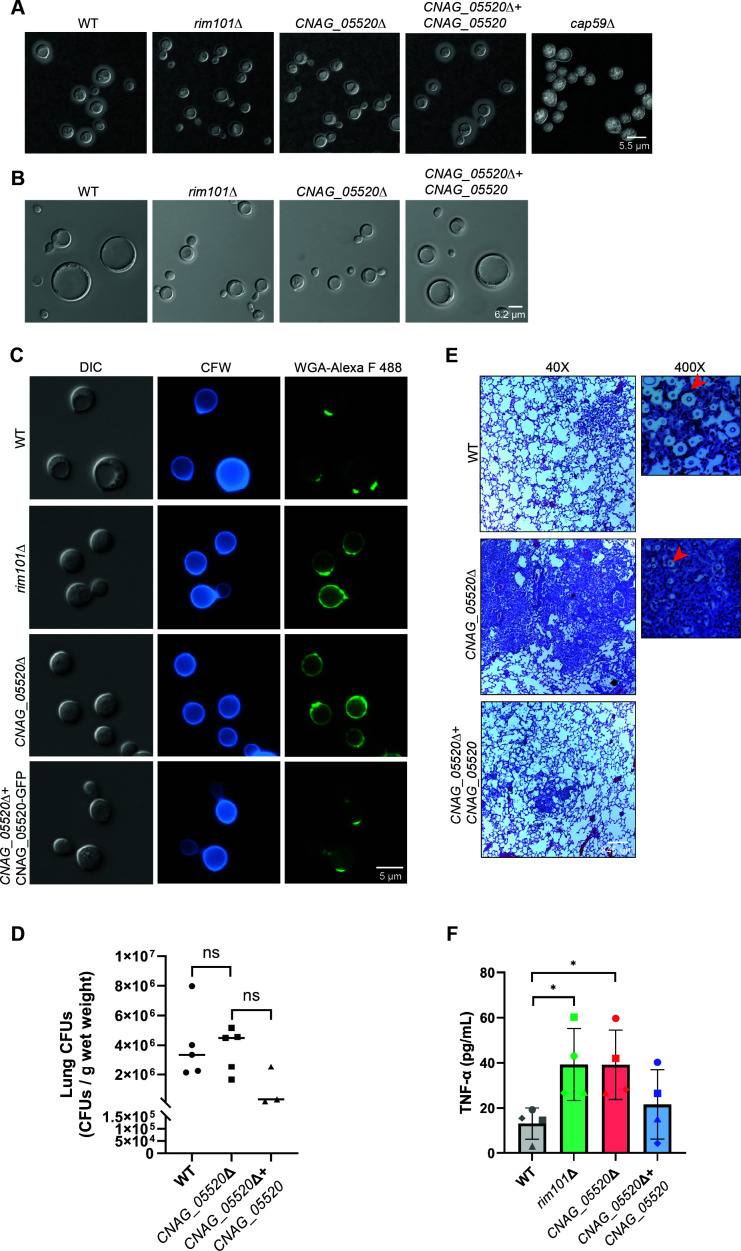
A *CNAG_05520*Δ mutant mimics the *rim101*Δ mutant virulence traits. (**A**) Capsule formation. Indicated strains were grown at capsule-inducing conditions (CO_2_-independent medium at 37°C for 3 days). Capsule formation was analyzed via India ink contrast staining and microscopy. Images were analyzed using ImageJ/Fiji. Representative DIC images are shown. (**B**) Titan cell formation. Indicated strains were grown overnight in YNB medium at 30°C and then transferred to *in vitro* titan cell-inducing conditions (OD_600_ of 0.001 in PBS + 10% HI-FBS) and then incubated for 24 hours at 37°C with 5% CO_2_. Cells were harvested and cell size assessed via microscopy. Images were analyzed using ImageJ/Fiji. Representative DIC images are shown. (**C**) Total chitin staining and exposure of cells grown under host-mimicking stress. Indicated strains were grown overnight in YPD medium and then transferred to CO_2_-independent medium (at an OD_600_ of 0.2) and grown for around 18 hours at 37°C. Cells were harvested and stained with calcofluor white (CFW—binds total cell wall chitin, blue channel) and Alexa Fluor 488 conjugated wheat germ agglutinin (WGA-Alexa F488—binds exposed cell wall chitin, GFP channel). Staining pattern and intensity were assessed via microscopy, and images were analyzed via ImageJ/Fiji. Representative DIC, blue channel (CFW staining), and GFP channel (WGA-Alexa F488) images are shown. (**D**) Lung fungal burden 7 days post-infection. Female A/J mice (5 mice per strain) were infected intranasally with 10^5^ cells/mouse of the indicated *C. neoformans* strains. Mice were sacrificed 7 days after infection, lungs harvested, and the fungal burden was assessed by quantitative culture. Data were plotted, and a Mann-Whitney U-test was done to assess statistical significance. (**E**) Histopathology analysis of infected murine lungs 7 days post-infection. Mice were infected as described above. Mice were sacrificed 7 days after infection, and lungs were fixed via intratracheal instillation with neutral buffered formalin under gravity flow. For visualization of lung pathology, lung sections were stained with hematoxylin and eosin. Red arrows show encapsulated yeast inside the inflamed lung tissue. (**F**) Tumor necrosis factor-α (TNF-α) was produced by murine bone marrow macrophages upon co-culturing with the indicated strains. Macrophages and fungal cells were co-cultured for 6 hours, and the supernatant was harvested. The secreted TNF-α was quantified with an enzyme-linked immunosorbent assay (ELISA). Student’s *t*-test was done to evaluate statistical significance (*P < 0.05).

We previously established that the *Cn* Rim pathway is required for efficient capsule production as well as titan cell formation (40). To determine if the *CNAG_05520* gene product might play a similar role in these pathogenesis-associated microbial phenotypes, we cultivated the WT strain, the *rim101*Δ and *CNAG_05520*Δ mutant, and a *CNAG_05520*Δ mutant complemented strain in capsule and titan cell-inducing conditions. We observed that cells lacking the *CNAG_05520* gene displayed similar capsule and titan cell formation defects as the *rim101*Δ mutant (Fig. 4A and B). Furthermore, like the *rim101*Δ mutant strain, the *CNAG_05520*Δ mutant strain displayed increased chitin exposure, as assessed by staining with Alexa Fluor 488-conjugated wheat germ agglutinin (WGA-Alexa F488) (Fig. 4C) (41).

Due to this altered cell wall polysaccharide exposure, Rim pathway mutants induce an excessive immune response in the infected lungs, resulting in failed immune evasion and early host death due to immune-mediated organ damage (40). To further assess phenotypic similarities between the *CNAG_05520*Δ mutant and the *rim101*Δ mutant, we assessed the degree of lung inflammation by histopathology analysis after an inhalational infection with either the WT, *CNAG_05520*Δ mutant, or *CNAG_05520* complemented strain at 7 days post-infection (Fig. 4D and E). A/J mice infected with the WT strain demonstrated an expected pattern of diffuse, minimal inflammatory infiltration of the infected lungs due to the potential for this microorganism to partially evade initial immune recognition. In contrast, infection with the *CNAG_05520*Δ mutant leads to a hyperinflammatory response in the lungs at 7 days post-infection, characterized by early and increased lung infiltration by neutrophils, despite similar fungal burdens (Fig. 4D and E). This observation is similar to what was previously observed in mice infected with a *Cn rim101*Δ mutant strain (21, 40). Additionally, we assessed production of the pro-inflammatory cytokine tumor necrosis factor-α (TNF-α) in bone marrow-derived macrophages harvested from CD1 mice in response to co-culturing with WT, *CNAG_05520*Δ mutant, *rim101*Δ mutant, or *CNAG_05520* complemented cells. In agreement with our histopathology results as well as previously published observations, more TNF-α was produced in response to co-culturing with the *CNAG_05520*Δ mutant and *rim101*Δ mutant cells compared to WT and *CNAG_05520* complemented cells (Fig. 4F) (40). These results indicate that defective capsule formation and altered cell wall organization due to defective Rim signaling result in increased exposure of epitopes recognized by host immune cells.

### The uncharacterized *CNAG_05520* gene product has similar functional motifs as the ascomycete PalF/Rim8 protein

**Fig 5:**
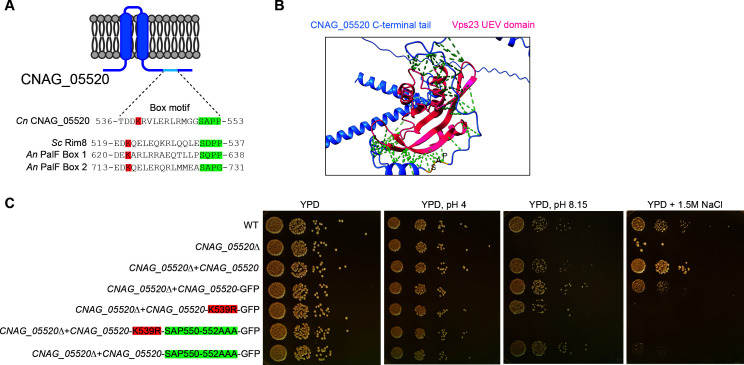
CNAG_05520 has similar protein features to the ascomycete Rim8 arrestin-like protein. (**A**) Model of predicted functional domains of the *Cn* CNAG_05520 protein, including two hydrophobic membrane-spanning domains and residues in the C-terminus similar to the Vps23-binding “box domain” of the Rim8/PalF proteins in *S. cerevisiae* (*Sc*) or *A. nidulans* (*An*). (**B**) AlphaFold model of potential interaction between *Cn* Vps23 and *Cn* CNAG_05520. The SxP motif is predicted to reside at the site of interaction. Distances of 5 Å or less are indicated with green dashed lines. (**C**) Growth phenotypes of strains with mutated CNAG_05520 lacking the SxP motif and ubiquitination site on alkaline and high salt stress-inducing plates. Indicated strains were incubated as serial spot dilutions for 72 hours at 30°C on YPD medium or on YPD medium either buffered to pH 8.15 or supplemented with 1.5 M NaCl.

The *CNAG_05520* gene encodes an uncharacterized protein with no clear homologs in ascomycete fungi. However, similar to the *Cn* Rra1 pH sensing protein, it shares a high degree of sequence similarity with genes present in the genomes of more closely related basidiomycete fungi when aligned with fungal genomes (Fig. S2). Unlike Rim8/PalF, the CNAG_05520 predicted protein does not have identifiable arrestin domains but two predicted membrane-spanning regions and several short motifs, which are homologous to functional motifs observed in Rim8/PalF proteins (25, 42). The CNAG_05520 protein contains four PxY motifs that typically bind the Rsp5 E3 ubiquitin ligase, similar to the three PxY motifs present in *Sc* Rim8 (25). Additionally, the CNAG_05520 protein contains a single lysine residue adjacent to two negatively charged amino acids in a C-terminal tail region, similar to the site of Rsp5-mediated ubiquitination in *Sc* Rim8. Notably, this lysine residue is predicted to be ubiquitinated by *Cn* Rsp5 by our above-described differential ubiquitination screen. Furthermore, the *CNAG_05520* gene product shares with *Sc* Rim8 a SxP motif 10-12 amino acids downstream from the predicted ubiquitinated lysine residue (42). This SxP motif, together with ubiquitin, is known as the box motif and is predicted to interact with the ubiquitin E2 variant (UEV) domain of an ESCRT-I protein (Fig. 5A). Computational modeling using the protein structure prediction artificial intelligence program, AlphaFold v. 3, predicts that the CNAG_05520 protein SxP motif is indeed in close proximity with the UEV domain of the *Cn* Rim pathway ESCRT-I scaffold protein Vps23 when interaction between these two proteins is modeled through the multimer mode of AlphaFold V. 3 (Fig. 5B). Furthermore, a CNAG_05520 mutant protein, with mutations of both the ubiquitinated lysine residue and the SxP motif, failed to functionally complement the *CNAG_05520*Δ mutant growth defects in the presence of high pH or 1.5 M NaCl (Fig. 5C). In contrast, in mutant strains expressing CNAG_05520 protein in which the ubiquitinated lysine residue or the SxP motif alone was mutated to arginine residues, the mutant strain growth defect is partially rescued in the presence of high pH or 1.5 M NaCl. These observations suggest functional relevance in CNAG_05520 of the predicted Vps23-interacting regions.

### The *CNAG_05520* gene product interacts with *Cn* Vps23

The *Sc* Rim8 arrestin protein interacts with Vps23, an early component of the ESCRT complex that facilitates recruitment and assembly of downstream Rim pathway proteins (25, 42). We, therefore, tested whether the CNAG_05520 protein similarly interacts with the *Cn* Vps23 protein. This interaction is predicted based on shared UEV-interaction motifs, present in both the CNAG_05520 protein and PalF/Rim8 proteins, that have been previously demonstrated to promote this protein-protein interaction (25, 42).

**Fig 6:**
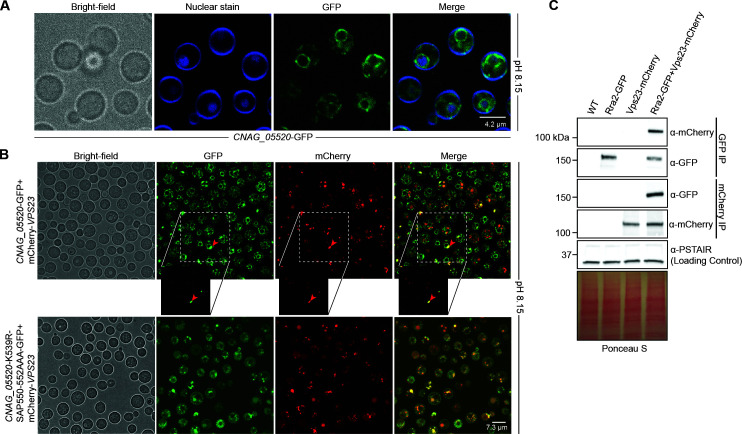
CNAG_05520 interacts with the Vsp23 ESCRT protein. (**A**) Localization of CNAG_05520-GFP at alkaline pH. The *CNAG_05520*Δ mutant strain expressing a GFP-tagged CNAG_05520 (*CNAG_05520-GFP*) was incubated for 1 hour in YPD medium buffered to pH 8.15. The location of the nucleus was assessed by staining with Hoechst nucleic acid stain. Protein localization was analyzed via confocal microscopy. Images were analyzed with ImageJ/Fiji. Representative Bright field and GFP channel images are shown. (**B**) Co-localization of CNAG_05520-GFP with mCherry-Vsp23 at alkaline pH. The *CNAG_05520*Δ and *vsp23*Δ mutant strains expressing a GFP-tagged CNAG_05520 and an mCherry-tagged Vsp23 (*CNAG_05520-GFP + mCherry-VSP23*) were incubated for 1 hour in YPD medium buffered to pH 8.15. Protein co-localization was analyzed via confocal microscopy. Images were analyzed via ImageJ/Fiji. Representative Bright field, GFP, and RFP channel images are shown with co-localization indicated with red arrows. (**C**) Co-immunoprecipitation of the GFP-tagged Rra2 protein and mCherry-tagged Vps23. The WT strain and the strains expressing *RRA2*-GFP, *VPS23*-mCherry, or both were incubated in YPD medium buffered to pH 8.15 for 1 hour at 30°C prior to fixing with 0.8% formaldehyde and NP-40-based protein extraction. Interaction of Rra2-GFP and Vps23-mCherry was analyzed via western blotting using an anti-GFP antibody (α-GFP) and anti-mCherry antibody (α-mCherry). Normalized cell lysates used as input for the immunoprecipitation assay were blotted in parallel and probed with anti-PSTAIR (α-PSTAIR) to assess even protein loading in addition to Ponceau staining.

These potential intermolecular interactions were further explored by assessing patterns of cellular localization of a CNAG_05520-GFP fluorescent fusion protein and a Vps23-mCherry fluorescent fusion protein using confocal microscopy. The CNAG_05520-GFP protein demonstrates prominent perinuclear localization, with focal regions of punctate enrichment both near the nucleus and at the cell surface (Fig. 6A). In the absence of the ubiquitinated lysine residue as well as the SxP motif, the perinuclear localization is lost (Fig. 6B). These surface puncta are similar to those observed in strains expressing the Rra1-GFP protein (21, 43). The Vps23-mCherry protein is enriched in punctate regions near the nucleus, and these structures largely co-localize with CNAG_05520-GFP (Fig. 6B). To confirm binding of *Cn* Vps23 to the CNAG_05520 gene product, we performed a co-immunoprecipitation assay with cells expressing both a CNAG_05520-GFP fluorescent fusion protein and a Vps23-mCherry fluorescent fusion protein. We were able to detect the GFP fluorescent protein fused to the CNAG_05520 gene product by probing with an anti-GFP antibody when Vps23-mCherry was immunoprecipitated, as well as the mCherry fluorescent protein fused to Vps23 with an anti-mCherry antibody when the CNAG_05520-GFP gene product was immunoprecipitated (Fig. 6C). This co-immunoprecipitation results complement our co-localization data and, together with our protein prediction modeling with AlphaFold and mutagenesis study, supports a model of conservation of function for CNAG_05520 as a Vps23-interactor.

### The *CNAG_05520* gene partially complements *Sc rim8*Δ mutant phenotypes

**Fig 7:**
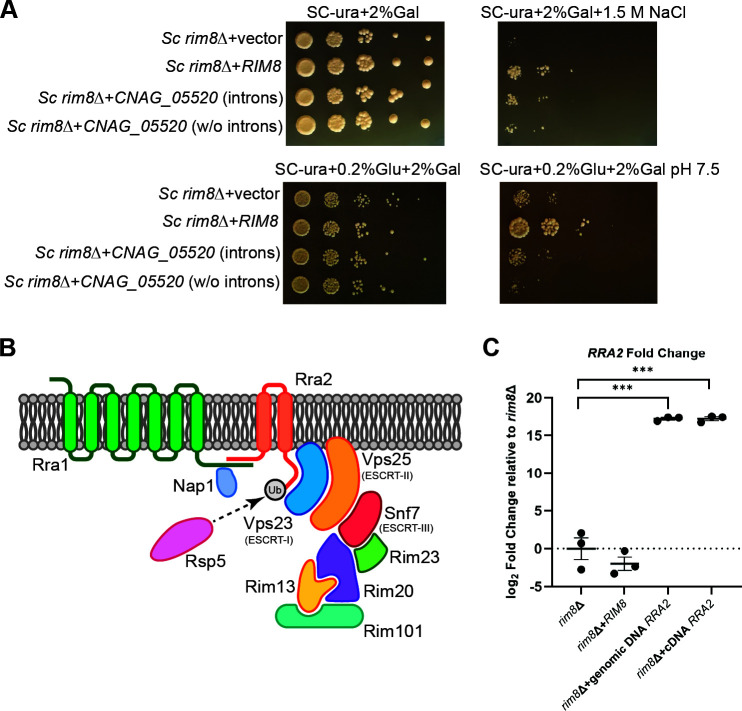
CNAG_05520 partially complements *rim8*Δ mutant growth phenotypes. (**A**) Cross-species complementation of the *S. cerevisiae* (*Sc*) *rim8*Δ high salt growth defect with *C. neoformans* (*Cn*) *CNAG_05520*. Indicated strains were incubated as serial spot dilutions for 72 hours at 30°C on synthetic complete medium (SC) without uracil and galactose as carbon source (SC-ura+Gal) or SC-ura+Gal medium supplemented with 1.5 M NaCl. (**B**) Transcript abundance of the *C. neoformans* (*Cn*) gene *RRA2* expressed in *Sc*. Indicated strains were incubated in SC medium at pH 7.5 for 1 hour at 30°C, and total RNA was harvested. Transcript abundance was assessed by qRT-PCR, and the log_2_ fold change relative to *Sc rim8*∆ was calculated using the ΔΔ C_T_ method. Transcript levels were normalized to *GPD1* transcript levels. The mean with error bars indicating the standard error of the mean of three biological replicates is plotted. Statistical analysis was performed using one-way ANOVA and an appropriate *ad hoc* test (***P < 0.0001). (**C**) Updated model of the *Cn* Rim pathway. The newly discovered CNAG_05520 protein functions as a linker between the Rim pathway activation complex and the downstream ESCRT complex. Rsp5 assists in Rim pathway activation via ubiquitination of CNAG_05520.

Although the *Sc* Rim8 protein and the *Cn* CNAG_05520 protein are divergent in primary sequence, they appear to serve orthologous roles in Rim pathway activation through interactions with ESCRT-I proteins. We therefore tested whether expression of *Cn CNAG_05520* in *S. cerevisiae* could partially or completely complement *Sc rim8*Δ mutant phenotypes. The *Cn CNAG_05520* gene was cloned into the 2µ pYES galactose-regulatable yeast expression vector and transformed into a *Sc rim8*Δ mutant. An intron-less complementary DNA (cDNA) version of this gene was also cloned into the same vector and transformed into the *Sc rim8*Δ mutant. Expression of the *Cn* CNAG_05520 gene in the *Sc rim8*Δ mutant cells was confirmed by real-time quantitative PCR (Fig. 7B). In contrast to a *rim8*Δ strain containing the empty pYES plasmid, the *rim8*Δ strain containing both the complete and the intron-less pYES-*CNAG_05520* plasmid strain displayed partial growth restoration on media containing 1.5 M NaCl (stress condition) and 2% galactose (drives gene expression in the pYES vector) (Fig. 7A). No phenotypic complementation was observed on galactose-containing media at pH 7.5 (Fig. 7A), even when 0.2% glucose was added to support growth. This partial functional complementation of a *Sc* Rim pathway mutant-associated phenotype by *Cn CNAG_05520* suggests functional convergence of CNAG_05520 and Rim8 despite sequence and evolutionary divergence.

Consistent with nomenclature for the previously identified *RRA1* gene (required for Rim pathway activation-1) (21), and to acknowledge the recent and independent identification of this gene through a large screen of *Cn* mutants for genes required for Rim pathway activity (38), the *CNAG_00520* gene was therefore named *RRA2*. The data presented here suggest that it serves to link Rra1 pH-sensing with downstream Rim pathway activation. Therefore, despite no identifiable sequence or structural similarities with the *Sc* Rim8 protein, it does share Rsp5-mediated ubiquitination and ESCRT-protein interactions that are common among Rim8/PalF proteins (Fig. 7C) (25–27).

## DISCUSSION

The fungal kingdom includes a vast array of ecologically diverse species that employ several unique molecular strategies to thrive in equally diverse habitats. Studying these stress-response pathways provides insight into evolutionary biology, but it also reveals novel cell biology that can be exploited in biotechnology or targeted by pharmaceuticals for the treatment of fungal infections. In this study, we explored the fungal-specific Rim alkaline pH response pathway, allowing acidophilic fungi to adapt to a pH above acidic levels. Although several downstream Rim pathway proteins are homologous among fungi across the ascomycota and basidiomycota phyla, differences occur in the upstream sensing complex (44).

External alkalinization is sensed by the seven transmembrane protein Rim21 in *Saccharomyces cerevisiae* and the homologous PalH in *Aspergillus nidulans* (19, 45). We have previously identified a sequence divergent, but functionally analogous, sensing protein in *Cryptococcus neoformans*, Rra1 (21). Although very different in primary amino acid sequence, there is evidence that the *Sc* Rim21 and the *Cn* Rra1 sensing proteins are both activated by alkaline pH-associated changes in the plasma membrane lipid bilayer asymmetry (19, 46). This model of pH sensing was supported by studies of the *Cn* Cdc50 lipid flippase regulatory subunit that was identified as a temporal effector of Rim pathway activation (47).

Containing seven predicted membrane-spanning domains, the *Sc* Rim21, *An* PalH, and *Cn* Rra1 pH sensor proteins resemble G-protein coupled receptors (GPCRs), although no interaction with G-proteins is known (21, 23). These GPCR-like sensor proteins might represent a growing group of seven-transmembrane domain-containing proteins with structural homology to GPCRs, but no known G protein binding partners (reviewed by reference 48). However, like GPCRs, *Sc* Rim21 and *An* PalH interact with arrestin-domain containing proteins that facilitate binding to ubiquitinating enzymes (23). While GPCRs typically associate with visual and β-arrestins, the Rim pathway sensor proteins associate with α-arrestin proteins, a distinct class of arrestins with PxY motifs to facilitate interaction with the WW domains of HECT E3 Ub ligases (49). Ubiquitination is best known for its function in the degradation of spent or misfolded protein, but it can also help direct changes in protein localization and function (50, 51). The specific ubiquitination events important in the *Sc* and *An* Rim pathway signal propagation involve the HECT E3 Ub ligase Rsp5 and its interaction with the arrestin domain-containing protein Rim8/PalF (23, 52). These events involve the ubiquitination of the *Sc* Rim8 arrestin upon binding to the Rim21 sensor protein, irrespective of the external pH (25). In contrast, ubiquitination of *An* PalF depends on sensor binding as well as an alkaline environment (23).

There are no proteins encoded in the *Cn* genome with a high degree of sequence similarity to *Sc* Rim8 or *An* PalF. We have previously identified four arrestin domain-containing proteins in *Cn* (Ali1, Ali2, Ali3, and Ali4), and subsequent phenotypic analysis of single and quadruple mutant strains suggests that none of these arrestin-like proteins are required for *Cn* Rim pathway activation.

Arrestin-mediated ubiquitination of the Rim8/PalF proteins by Rsp5 does not result in its accelerated protein degradation, but rather in the addition of a ubiquitin moiety, which likely serves as an interface for binding of the UEV domain of the ESCRT-I protein Vps23 (42). Proteins of the ESCRT complex are typically involved in the sorting of proteins to lysosomes or the vacuole for degradation (reviewed in reference 53). However, in this instance, the ESCRT complex has an established role in the fungal Rim/Pal pathway to recruit the Rim13/PalB protease to activate the Rim101/PacC transcription factor and to potentially internalize the sensor proteins for their regulated degradation. In addition to protein sorting to the vacuole/lysosomes, the only other known function of Vps23 is to serve as a linker between the Rim8/PalF proteins and the ESCRT-II protein, a role for which it was likely co-opted (52).

We have previously demonstrated that ESCRT proteins are also required for *Cn* Rim pathway activation (21). However, the steps linking surface pH recognition and ESCRT assembly have not been previously identified in this pathogenic fungus. Our data have identified Rra2 as a target of Rsp5 and as a protein required for *Cn* Rim pathway activation. Although Rra2 lacks distinct sequence similarity to Rim8, it shares similar protein features, including a ubiquitinated lysine residue 11 to 13 amino acid residues upstream of an SxP motif in the C-terminal region of these proteins. We also demonstrate its co-localization and co-immunoprecipitation with Vps23, strongly supporting that it serves as an Rsp5 adapter protein for Rim pathway activation. Molecular modeling of potential structural interactions between *Cn* Vps23 suggests similar sites of interaction, as has been experimentally verified between *Sc* Rim8 and *Sc* Vps23 (25). A recent study conducted by Madhani et al. suggests functional interaction between the membrane pH sensor Rra1 and the newly identified Rim1-pathway component Rra2 (38). Based on their findings as well as our findings here, we therefore suggest that Rra2 acts as a linking component between the Rra1 pH membrane sensor and the Rim101-activating ESCRT complex. Our data further suggest that, similar to *Sc* Rsp5, *Cn* Rsp5 regulates Rim pathway activation by ubiquitination of the linker protein Rra2 (Fig. 7A).

Our data presented here demonstrate that Rra2, like other Rim pathway proteins, is essential for Rim pathway-dependent adaptation of this fungal pathogen to the alkaline pH environment within the infected host. These adaptations include host-mediated cell surface changes that provide efficient capsule attachment and immune avoidance. In the absence of Rra2, *Cn* fails to maintain its surface capsule and does not make titan cells in host-like *in vitro* growth conditions. Additionally, the *rra2*Δ mutant induces an altered pattern of early inflammatory responses in the infected lung. Similar patterns of excessive early inflammatory responses are observed in other *Cn* Rim pathway mutants, resulting in immune-mediated pathology and accelerated mortality (40). Together, these data demonstrate a central role for this non-traditional Ub ligase adaptor in *Cn* Rim signaling.

The *Cn* Rsp5 E3 Ub ligase regulates several stress response processes in addition to adaptation to alkaline pH (28). Moreover, this ubiquitinating enzyme uses both arrestin-like proteins as well as proteins with no discernible arrestin domains as adaptors to bind its target proteins (28, 33). There is considerable interest in ubiquitination as a therapeutic target for many conditions. These include many cancers and infections by eukaryotic pathogens (reviewed in references 54, 55). Importantly, many of the proteins involved in the Rim pathway interactions are either only found in fungi or contain fungal-specific interacting domains (56). Our results here, therefore, suggest that fungal-specific inhibitors of Rsp5-mediated ubiquitination might be harnessed to modulate microbial survival *in vivo*.

## MATERIALS AND METHODS

### Strains, media, and growth conditions

A list of the strains used in this study is given in Table S5, including details of strains obtained from Dr. Hiten Madhani (39). The *C. neoformans* var. *grubii* H99 (*MAT*α) strain background was used as wild type (WT) for this study unless otherwise specified (57). All strains were stored in glycerol stocks at −80°C and were recovered on YPD medium (1% yeast extract, 2% peptone, 2% dextrose, and 2% agar for solid medium) before each experiment. Unless otherwise indicated, strains were incubated in YPD medium at 30°C with shaking at 150 rpm. Complemented mutant strains and fluorescent-tagged strains were constructed using CRISPR-Cas9 gene editing techniques as previously described (28). These constructs were integrated into the *Cn* genome at the Safe Haven site following targeting with a single guide RNA oligonucleotide with a GGTCTGATCAGTTATAGACG binding site (58). To obtain strains of the opposite mating type, the *MAT*α *rra2*Δ mutant strain (MDP80) and the *MAT*α *VPS23-*mCherry expressing strain (KS272) were crossed with the KN99a strain to obtain *MAT*a strains (MDP87 and MDP93, respectively), following the approach described earlier (28). The *MAT*a *rra2*Δ (MDP87) and *MAT*a *rsp5*Δ (MDP11) mutant strains were crossed with the KS208 strain expressing GFP-tagged *RIM101*, yielding MDP81 (*rsp5*Δ expressing *GFP-RIM101*) and MDP82 (*rra2*Δ expressing *GFP-RIM101*). Similarly, the *MAT*a *rsp5*Δ (MDP11) mutant strain was crossed with the KS161 strain expressing the constitutively active truncated *RIM101* gene under transcriptional control of the *GAL7* promoter to obtain strain MDP47, an *rsp5*∆ strain expressing truncated *RIM101*. Primer pair AA5778 and AA5779 were used to amplify the *CNAG_05520* open reading frame (ORF) as well as 600 bp upstream and downstream for integration with the pSDMA57 plasmid to create the *rra2*Δ+*RRA2* complemented strain (58). The plasmid was linearized with primer pair AA5780 and AA5781 with overlaps to the *CNAG_05520* fragment for assembly with the NEBuilder HiFi DNA Assembly kit (NEB), as previously described (28). In a similar manner, the primer pair AA5754 and AA5755 were used to amplify the *CNAG_05520* ORF, and primer pair AA5752 and AA5753 the pGWKS10 plasmid containing the clover (GFP) fluorescent gene for assembly with the NEBuilder HiFi DNA Assembly kit to create the GFP-tagged *RRA2* expressing strain (59).

Overlapping primer pair AA5782 and AA5783 was used to introduce the K539R mutation into the *CNAG_05520* ORF in the pGWKS10 plasmid. The SAP550-552AAA mutation was introduced using the primer pair AA5817 and AA5755 to create a megaprimer that was used in a subsequent PCR to amplify the whole *CNAG_05520* ORF for assembly into the pGWKS10 plasmid, an approach described earlier (60). The GFP-tagged *RRA2*-expressing strains were crossed with the MDP93 *VPS23-mCherry*-expressing strain to obtain strains with both tagged genes.

The pYES2.1 plasmid containing the WT *Sc RIM8* gene was constructed using the primer pair AA5862 and AA5863 to amplify the *RIM8* gene from *Sc* BY4741 genomic DNA. The primer pair AA5864 and AA5865 was used to amplify the pYES2.1 plasmid with overlapping regions to the *RIM8* gene for assembly with the NEBuilder HiFi DNA Assembly kit. The primer pair AA5858 and AA5859 was used to amplify the *CNAG_05520* ORF from *Cn* WT genomic DNA (with introns) or cDNA (without introns), and primer pair AA5860 and AA5861 was used to amplify the pYES2.1 plasmid with overlapping regions to the *CNAG_05520* ORF. These two fragments were assembled as before using the NEBuilder HiFi DNA Assembly kit. The *Sc* pYES2.1 plasmids were transformed into *Sc rim8*Δ mutant cells with the lithium acetate approach described in the pYES2.1 TOPO TA Yeast Expression Kit protocol (Thermo Fisher Scientific). Strains were verified with diagnostic PCR and Sanger sequencing. Oligonucleotide primers used for strain validation and construction are listed in Table S6.

Capsule induction was performed by incubating WT, *rra2*Δ mutant, *rim101*Δ mutant, and *cap59*Δ mutant (acapsular control strain) cells in CO_2_-independent medium (CIM; Gibco) at 37°C while shaking for 72 hours and then staining with India ink before microscopy imaging (Zeiss Axio Imager A1) (28).

To induce titanization, cells were precultured for 18 hours in YNB at 30°C. Cells were then washed 1× with phosphate-buffered saline (PBS) and transferred to 10 mL PBS + 10% heat-inactivated fetal bovine serum (HI-FBS) in 6-well tissue culture plates. For low-density growth (titan induction), cells were inoculated to an OD_600_ of 0.001 and incubated for 72 hours in a tissue culture incubator at 37°C with 5% CO_2_. Cells were then imaged with microscopy as before (61).

To assess cell wall integrity through the extent of chitin exposure, WT cryptococcal cells, *rra2*Δ mutant, *rim101*Δ mutant, as well as *rra2*Δ+*RRA2* complemented cells, were grown for 18 hours at 30°C in YPD medium before being shifted to fresh YPD medium for 7 hours at 30°C. Cells were harvested, standardized to an OD_600_ of 0.25, resuspended in CO_2_-independent medium, and incubated at 37°C for 18 hours. Cells were harvested and stained with 100 µg/mL Alexa F488-conjugated wheat germ agglutinin (WGA; Molecular Probes) for 35 minutes, followed by 25 µg/mL calcofluor white (CFW; Fluka Analytical) for 10 minutes, and finally imaged with microscopy, as described before (62).

Survival in the presence of stress requiring intact Rim pathway activation was assessed through a spot-dilution assay on supplemented YPD medium as previously described (21). Fungal cells were serially diluted and spotted onto solid media plates and exposed to alkaline pH (disrupts electrochemical gradient) and sodium chloride (NaCl; provides osmotic/ionic stress). To assess *S. cerevisiae rim8*Δ mutant cell growth—as well as the growth of *rim8*Δ mutant cells complemented with intact *RIM8*, *RRA2* including introns, or *RRA2* without introns—on stress-inducing plates, synthetic complete (SC) medium was used, lacking uracil and with 2% galactose as carbon source. In alkaline solid medium plates with 2% galactose, 0.2% glucose was added to support the initial growth of complemented mutant strains.

### Real-time quantitative PCR

To quantify the expression levels of the *CIG1* gene, under the transcriptional regulation of the Rim101 transcription factor, WT, *rsp5*Δ mutant, *sre1*Δ mutant, *rim101*Δ mutant, and *rra2*Δ mutant cells were grown for 18 hours at 30°C in YPD medium. Cells were harvested and resuspended in YPD medium buffered to pH 8.15 and incubated for 1 hour at 30°C. Cells were collected and RNA was extracted following the protocol of the Qiagen RNeasy Plant Minikit (Qiagen). cDNA was prepared by reverse-transcriptase PCR using the AffinityScript cDNA qPCR Synthesis kit (Agilent Technologies), following the included protocol. Real-time quantitative PCR was performed as described before, using the described primers for *CIG1* cDNA amplification (47). Similarly, *Sc rim8*Δ mutant cells containing an empty pYES 2.1 vector, a pYES 2.1 vector containing WT *RIM8,* or *Cn RRA2* with or without introns were grown for 18 hours at 30°C in SC medium without uracil and with 2% galactose. Cells were harvested and resuspended in SC medium buffered to pH 7.5 and incubated for 1 hour at 30°C. Cells were collected and RNA was extracted as before. Complementary DNA was prepared as before, and real-time quantitative PCR was performed as described before, using primer pair AA5924 and AA5925 for *RRA2* cDNA amplification.

### Histology and persistence in a murine model

We used the murine inhalation model of cryptococcosis (28) to assess fungal survival and host response after lung infection with WT cryptococcal cells, as well as with *rra2*Δ mutant and *rra2*Δ+*RRA2* complemented cells. For each strain, five female A/J mice were used (Charles River Laboratories). Mice were anesthetized by isoflurane inhalation and intranasally inoculated with 1 × 10^5^ fungal cells of PBS-washed overnight cultures (18 hours) of the indicated strains. Mice were sacrificed at 7 days post-infection, and lungs were harvested, weighed, and homogenized in cold PBS. Colony-forming units (CFU) were calculated through quantitative culturing and were represented as CFU/gram lung wet weight. For histopathology at 7 days post-infection, mice were sacrificed using a lethal dose of pentobarbital sodium, and lungs were fixed via intratracheal instillation of neutral buffered formalin under gravity flow (63). Fixed lungs were submitted to the Immunohistopathology Core at Duke University for paraffin embedding and staining with hematoxylin and eosin. Brightfield microscopy images were visualized with a Zeiss Axio Imager A1 microscope (using 4× or 40× objectives) to assess the distribution of the yeast cells and the immune response. Images were taken with an AxioCam MRm digital camera with ZEN Pro software (Zeiss).

### Preparation of bone marrow-derived macrophages and *in vitro* co-culturing with cryptococcal cells

Bone marrow cells were derived from male CD1 mice purchased from Charles River Laboratories. Murine bone marrow cells were prepared and co-cultured with cryptococcal cells as described previously (14, 64). This included the isolation of femurs from mice and flushing bone marrow with 10 mL cold PBS using a 27½ gauge needle. Red blood cells were lysed in 1× red blood cell lysis buffer (0.15 M NH_4_Cl, 1 mM NaHCO_3_, pH 7.4) for 5 minutes and cells were resuspended in 1× Dulbecco’s modified Eagle’s medium (DMEM; +4.5 g/L D-glucose, +L-glutamine, +110 mg/L sodium pyruvate) with 1 U/mL penicillin/streptomycin with 10% (vol/vol) HI-FBS and 3 ng/mL recombinant mouse GM-CSF (BioLegend) for differentiation at a cell concentration of 2.5 × 10^5^ cells/mL in 150 × 15 mm petri plates at 37°C with 5% CO_2_. The media was refreshed after 4 days, and the cells were harvested on day 7.

For co-culturing, *C. neoformans* cells were washed 2× with PBS, counted, and added to bone marrow macrophages in wells of a 96-well plate at a concentration of 5 × 10^5^ fungal cells per well (10:1 *C*. *neoformans* cells:bone marrow macrophages). Co-cultures were incubated for 6 hours at 37°C with 5% CO_2_. Supernatants were collected and stored at −80°C until analysis. Secreted TNF-α was quantified in supernatants by enzyme-linked immunosorbent assay (ELISA; BioLegend). Three replicates were analyzed for each *Cn* strain.

### Differential ubiquitination experiment and analysis

The WT strain (*n* = 3) and the *rsp5*Δ mutant strain (*n* = 3) were incubated in liquid YPD medium for 18 hours. Cultures were normalized to an OD_600_ of 3 in fresh YPD medium and fresh YPD medium buffered to pH 4 and pH 8.15 and conditioned for 1 hour. Samples were washed twice with PBS and flash frozen on dry ice. Sample preparation and mass spectrometry analysis were performed by the Duke Proteomics Core Facility as previously described (28). Differentially ubiquitinated peptides were plotted using the ggplot package in the R coding language using RStudio. We calculated fold changes between the peptides identified from the different strains based on the intensity values and calculated a two-tailed heteroscedastic *t*-test on log_2_-transformed data for each of these comparisons. We then indicated if a particular peptide was increasing or decreasing with a *P*-value of 0.05 and fold change of 1.5 between the different strains. Mutants available in a mutant collection lacking the equivalent proteins ubiquitinated by *Cn* Rsp5, as identified through the differential ubiquitination assay, were then assessed for growth defects in the presence of alkaline pH and a high salt concentration, as well as the inability to induce WT levels of *CIG1* expression.

### Protein purification and immunoblotting

The WT strain, the *rsp5*Δ mutant strain, the *rim101*Δ mutant strain, and the *rra2*Δ mutant strain were incubated in liquid YPD medium for 18 hours. Cultures were washed twice in PBS and subsequently normalized by OD_600_. Cells were conditioned in fresh YPD medium and fresh YPD medium buffered at pH 4 or pH 8 for 1 hour. To collect total cell lysates, cultures were pelleted, flash frozen on dry ice, and lysed by bead beating. Lysates were collected in 20% trichloroacetic acid (TCA) buffer and processed for western blotting as described before (61). Western blot membranes were stained with Ponceau S stain and then probed with an anti-GFP monoclonal primary antibody (1/5,000 dilution; Roche Applied Science, 11814460001; lot 14717400) and an anti-mouse peroxidase-conjugated secondary antibody (1/25,000 dilution; Jackson ImmunoResearch Laboratories, Inc., 111-035-008; lot 128022), before detection by enhanced chemiluminescence using SuperSignal West Pico (Thermofisher).

The WT strain (H99) and all strains expressing GFP-tagged *RRA2,* mCherry-tagged *VPS23*, or both tagged genes were incubated in liquid YPD medium for 18 hours. The strains were then normalized by OD_600_ and conditioned in YPD medium buffered to pH 8.15 for 1 hour. The cells were then fixed with 0.8% (vol/vol) formaldehyde to crosslink protein interactions and quenched with 125 mM glycine. Total cell lysates were prepared as before, and normalized cell lysates were immunoprecipitated through the addition of 25 µL GFP-TRAP or RFP-TRAP agarose bead suspension (ChromoTek) equilibrated in NP-40 lysis buffer, as described previously (21). Lysates were pre-cleared with control agarose beads for 1 hour, and GFP-TRAP and RFP-TRAP agarose beads were blocked with 30 mg/mL bovine serum albumin in NP-40 lysis buffer for 1 hour. Protein eluted from the agarose beads was added to LDS loading buffer and incubated at 95°C for 5 minutes. Western blotting was performed as earlier. To detect precipitation of fluorescent protein, blots were incubated with an anti-GFP monoclonal primary antibody (1/4,000 dilution; Roche Applied Science, 11814460001; lot 14717400) or an anti-red fluorescent protein (anti-RFP) polyclonal primary antibody (1:4,000 dilution, Abcam, ab124754) overnight. An anti-mouse peroxidase-conjugated secondary antibody (1/25,000 dilution; Jackson ImmunoResearch Laboratories, Inc., 111-035-008; lot 128022) was used to allow detection by enhanced chemiluminescence when probing with anti-GFP and an anti-rabbit peroxidase-conjugated secondary antibody (1/25,000 dilution; Jackson ImmunoResearch Laboratories, Inc., 115-035-174; lot 127837 when probing with anti-RFP. A control western blot was performed in parallel with aliquots of normalized cell lysates used as input for the immunoprecipitation experiments. This blot was probed with an anti-PSTAIR monoclonal primary antibody (1/4,000 dilution; Sigma, P7962; lot 015M4840V) and an anti-mouse peroxidase-conjugated secondary antibody as before.

### Microscopy

Strains expressing GFP-tagged *RIM101* were incubated in liquid YPD medium for 18 hours at 30°C while shaking. Cells were harvested and resuspended in YPD medium adjusted to pH 8.15 and incubated for a further 1 hour at 30°C while shaking. Cells were collected and stained with Hoechst 33342 live nuclear stain for 10 minutes, washed with PBS, and imaged immediately. Differential interference microscopy (DIC) and fluorescent images were visualized with a Zeiss Axio Imager A1 fluorescence microscope (60× or 100× objectives) using a GFP filter for GFP fluorescence. Images were taken with an AxioCam MRm digital camera with ZEN Pro software (Zeiss). The same exposure time was standardized across all strains, and images were analyzed using ImageJ/Fiji software (65).

To obtain high-resolution images of GFP-tagged *RRA2* and mCherry-tagged *VPS23*-expressing cells, we employed confocal microscopy. Cells were prepared as for fluorescence microscopy, and 4 µL of cell suspension was placed in an ibidi 8-well glass bottom µ-slide and covered with a 200 µL slab of solidified 2% agarose (Thermo Fisher Scientific). Specific cell density was adjusted to allow for single-cell monolayers for optimal imaging. Imaging was conducted at room temperature (21°C to 23°C). Images were acquired with a Nikon Ti2-E stand equipped with a Yokogawa CSU-W1 spinning disk confocal unit and a 100×/1.35 silicone oil objective. Images were captured using a Hamamatsu ORCA-Fusion BT sCMOS camera controlled by NIS-Elements software (Nikon Instruments). To capture the entire volume of the cell, 17 Z-slices were acquired at 0.48 µm steps. Cells expressing GFP-tagged *RRA2* were imaged at 25% laser power, and mCherry-tagged *VPS23*-expressing cells at 75% laser power. The exposure time for all confocal images was 400 milliseconds. Images were processed with ImageJ/Fiji software (65).

### Phylogenetic tree construction and *in silico* modeling

*Cryptococcus neoformans* Rra1 and Rra2 protein sequences were searched against the NCBI GenBank database using a BLASTP search with default settings. Multiple sequence alignments were generated from the top 100 significant BLASTP search hits using Clustal Omega hosted by EMBL-EBI with default settings (66). Phylogenetic trees were constructed using aligned protein sequences as well as the *S. cerevisiae* Rim8 and *A. nidulans* PalF protein sequences using IQ-TREE v. 2 and were visualized with iTOL v. 7 (67, 68).

To model potential interactions between the *Cn* Rra2 protein and *Cn* Vps23, Google DeepMind AlphaFold v. 3 was used (69). Full protein sequences were uploaded to the AlphaFold server and processed using the default settings. The results, including the predicted aligned error plot, were visualized with ChimeraX, and distances between the two proteins of 5 Å or less were determined (70).

## Data Availability

Data obtained through the differential ubiquitination screen is provided in the supplemental material. All other data are available on request.

## SUPPLEMENTAL MATERIAL

Supplemental Figure Legends mbio.00732-25-s0001.docx Legends for Figures S1 to S3.

Figure S1 mbio.00732-25-s0002.tif *Cryptococcus neoformans* arrestin mutants have no growth defects at alkaline pH or high salt.

Figure S2 mbio.00732-25-s0003.tif Phylogenetic trees illustrating the divergence of the *Aspergillus niger* and *Saccharomyces cerevisiae* Rim21, Rim8, PalH, and PalF from the basidiomycete equivalent proteins.

Figure S3 mbio.00732-25-s0004.tif The predicted alignment error plot of an AlphaFold model of potential interaction between Cn Vps23 and Cn CNAG_05520.

Supplemental Tables mbio.00732-25-s0005.xlsx Tables S1 to S6.

ASM does not own the copyrights to Supplemental Material that may be linked to, or accessed through, an article. The authors have granted ASM a non-exclusive, world-wide license to publish the Supplemental Material files. Please contact the corresponding author directly for reuse.
