# Supplementary material for: Rsp5-mediated ubiquitination of a functional analog of the Rim8 arrestin facilitates Rim pathway activation in Cryptococcus neoformans
Source: mBio. 2025 Jul 22;16(8):e00732-25. doi: 10.1128/mbio.00732-25 (PMC12345238; doi:10.1128/mbio.00732-25)
Supplement: Supplemental Figure Legends — Legends for Figures S1 to S3. [file mbio.00732-25-s0001.docx]

**Figure S1. The arrestin-like Ali1, Ali2, Ali3, and Ali4 proteins are not required individually or collectively for growth at alkaline pH**. Indicated strains were incubated for 72 hours as serial spot dilutions on YPD medium and YPD medium buffered to pH 8.15.

**Figure S2. Rra1 and Rra2 are basidiomycete specific proteins.** Phylogenetic tree of the fungal Rra1 (**A**) and Rra2 (**B**) proteins. *C. neoformans* Rra1 and Rra2 protein sequences were searched against NCBI Genbank database using BLASTP search with default settings. Multiple sequence alignments (MSA) were generated from the top 100 significant BLASTP search hits using Clustal Omega hosted by EMBL-EBI with default settings. Phylogenetic trees were constructed using aligned protein sequences as well as the *S. cerevisiae* Rim8 and *A. nidulans* PalF protein sequences using IQ-TREE v. 2 and were visualized with iTOL v. 7.

**Figure S3.** The predicted alignment error plot of an AlphaFold model of potential interaction between *Cn* Vps23 and *Cn* CNAG_05520. The SxP motif is predicted to reside at the site of interaction. The plot is shown with close interaction between the two proteins highlighted with red boxes.
