# Supplementary figures and images for: Rsp5-mediated ubiquitination of a functional analog of the Rim8 arrestin facilitates Rim pathway activation in Cryptococcus neoformans
Source: mBio. 2025 Jul 22;16(8):e00732-25. doi: 10.1128/mbio.00732-25 (PMC12345238; doi:10.1128/mbio.00732-25)

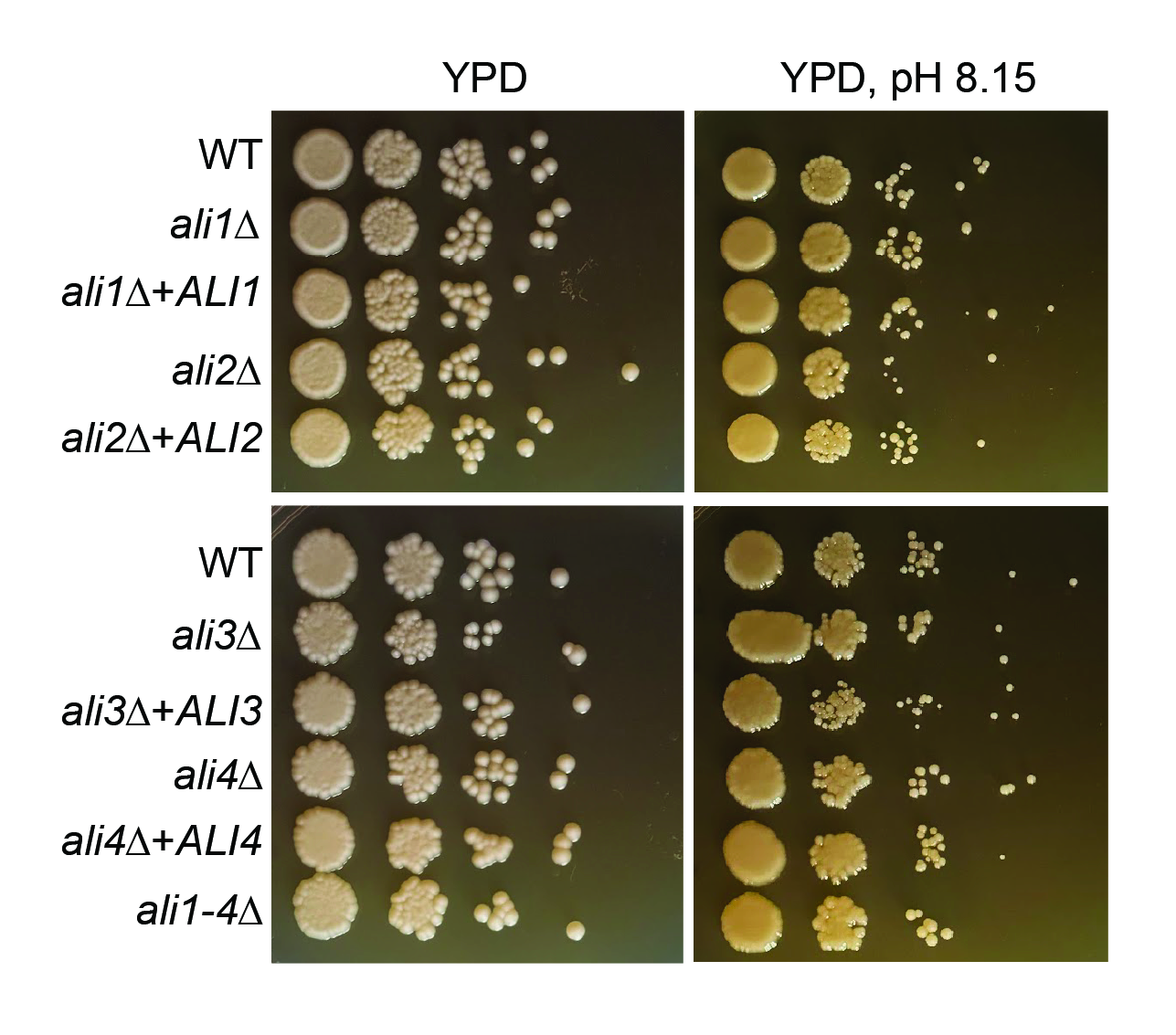

Supplement: Figure S1 — Cryptococcus neoformans arrestin mutants have no growth defects at alkaline pH or high salt. [file mbio.00732-25-s0002.tif]

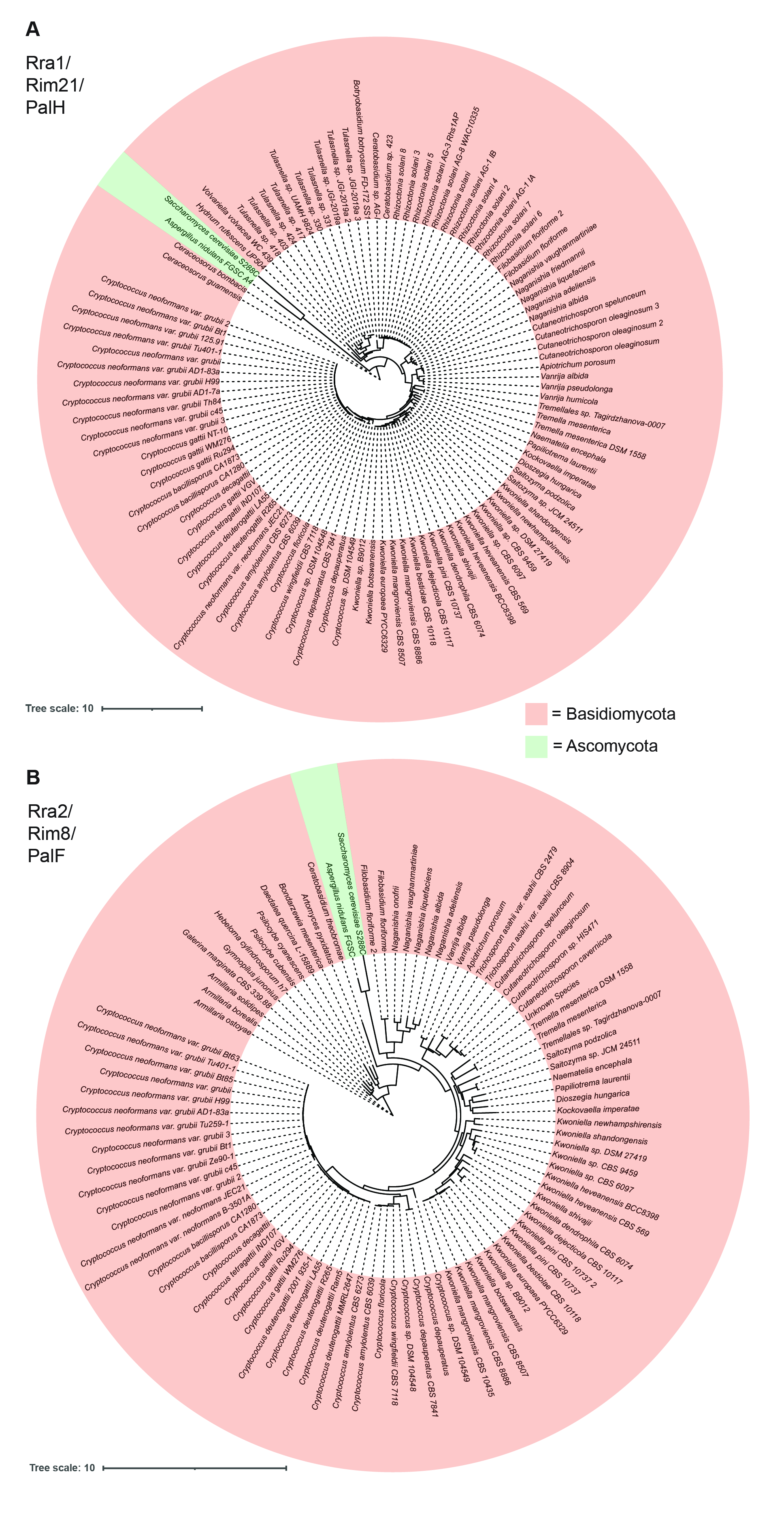

Supplement: Figure S2 — Phylogenetic trees illustrating the divergence of the Aspergillus niger and Saccharomyces cerevisiae Rim21, Rim8, PalH, and PalF from the basidiomycete equivalent proteins. [file mbio.00732-25-s0003.tif]

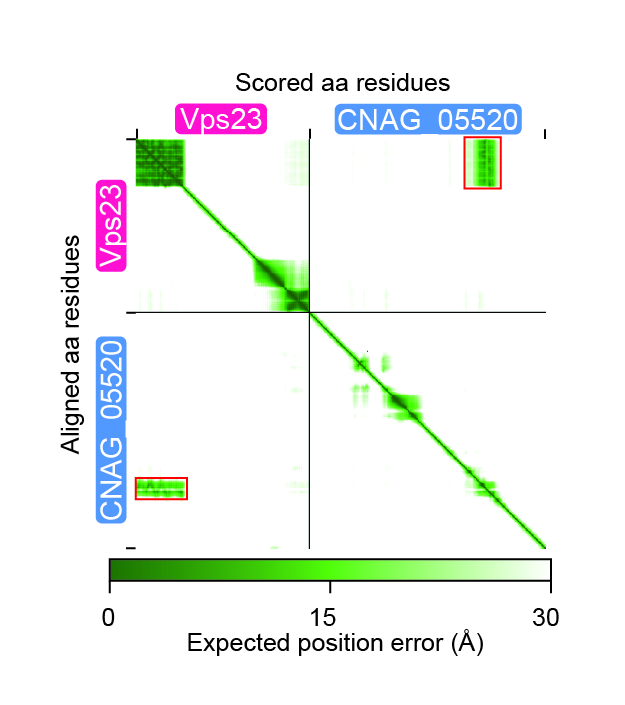

Supplement: Figure S3 — The predicted alignment error plot of an AlphaFold model of potential interaction between Cn Vps23 and Cn CNAG_05520. [file mbio.00732-25-s0004.tif]
